# Skeletal Class III Malocclusion Is Associated with *ADAMTS2* Variants and Reduced Expression in a Familial Case

**DOI:** 10.3390/ijms231810673

**Published:** 2022-09-14

**Authors:** Siyue Yao, Xi Zhou, Barbara Vona, Liwen Fan, Chengcheng Zhang, Dandan Li, Hua Yuan, Yifei Du, Lan Ma, Yongchu Pan

**Affiliations:** 1 Department of Orthodontics, The Affiliated Stomatology Hospital of Nanjing Medical University, Nanjing 210000, China; 2 Jiangsu Province Key Laboratory of Oral Diseases, Nanjing Medical University, Nanjing 210000, China; 3 The Affiliated Stomatology Hospital of Suzhou Vocational Health College, Suzhou 215000, China; 4 Institute of Human Genetics, University Medical Center Göttingen, 37073 Göttingen, Germany; 5 Institute for Auditory Neuroscience and Inner Ear Lab, University Medical Center Göttingen, 37075 Göttingen, Germany; 6 Department of Oral and Maxillofacial Surgery, The Affiliated Stomatology Hospital of Nanjing Medical University, Nanjing 210000, China; 7 Jiangsu Province Engineering Research Center of Stomatological Translational Medicine, Nanjing Medical University, Nanjing 210000, China

**Keywords:** malocclusion, craniofacial abnormalities, whole exome sequencing, genetic research, zebrafish, developmental biology

## Abstract

Skeletal Class III malocclusion with maxillary deficiency is a severe maxillofacial disease with unclear pathogenic mechanisms. We recruited a Han Chinese family who was clinically diagnosed with skeletal Class III malocclusion and maxillary deficiency. Using whole exome sequencing, a missense variant in *ADAMTS2* (NM_014244: c.3506G>T: p.G1169V) was identified and predicted as deleterious by in silico tools. We also found *ADAMTS2* variants associated with deficient maxillary development in a cohort. *ADAMTS2* expression in HEK293 cells showed significant decrease due to the variant, which was also consistent in dental pulp stem cells from the proband and a healthy control. In the *adamts2*-knockdown zebrafish model, the length and width of the ethmoid plate, as well as the length of the palatoquadrate became significantly shorter than the control group (*p* < 0.001), while there was no significant difference in the length and width of the mandible. The expression of Sox3, which was required in early embryonic craniofacial development, was significantly downregulated in the *adamts2*-knockdown zebrafish embryos. Bioinformatic and cellular studies showed that the decreased expression of ADAMTS2 may inhibit downstream ErbB signaling pathway transduction and restrain subsequent osteogenesis in human adult mesenchymal stromal cells. Collectively, these data showed that *ADAMTS2* (c.3506G>T: p.G1169V) may confer susceptibility to risk of skeletal Class III malocclusion with maxillary deficiency.

## 1. Introduction

Skeletal Class III malocclusion is a severe maxillofacial disorder characterized by maxillary deficiency and/or mandibular prognathism, which can occur as part of a syndrome or as an isolated trait [1]. The concave profile and crossbite have considerable impacts on chewing function and psychological aspects of patients’ lives [2]. Currently, most adult patients with severe skeletal Class III malocclusion require orthognathic surgery to obtain a good facial shape and proper bite [3]. According to a survey conducted by the Chinese Stomatological Association in 2000, the incidence of Class III malocclusion in the Chinese population was 14.94% for primary dentition, 9.65% for alternate dentition, and 14.98% for permanent dentition [4]. The etiology of skeletal Class III malocclusion is multifactorial, including environmental and genetic factors. Some pathological factors such as enlarged tonsils, and bad habits like chronic mouth breathing are associated with skeletal Class III malocclusion [5,6].

Both mandibular prognathism and/or maxillary deficiency are features of skeletal Class III malocclusion, but they differ in bone formation patterns [7,8]. In recent decades, several susceptibility genes like *EPB41*, *MYO1H*, *COL2A1*, *TGFβ1*, and *FGFR2* have been proven to play important roles in the etiology of skeletal Class III malocclusion with mandibular prognathism [9]. However, only a few studies have focused on skeletal Class III malocclusion with maxillary deficiency. Oh et al. identified a region around D12Mit7 on mouse chromosome 12 that determined maxillary growth [10]. Nikopensius et al. demonstrated that *DUSP6* may be a candidate gene related to maxillary deficiency by whole exome sequencing (WES) [11]. These studies greatly expand the molecular genetic understanding of skeletal Class III malocclusion, but further studies are still needed for better understanding of its mechanism.

In the present study, we explored the association between skeletal Class III malocclusion with maxillary deficiency and genetic variants in a Chinese Han family and cohort. Phenotype replication was carried out in zebrafish embryo models to further evaluate the potential biological functions of the susceptibility gene.

## 2. Results

### 2.1. ADAMTS2 Is Associated with Maxillary Development

**Figure 1:**
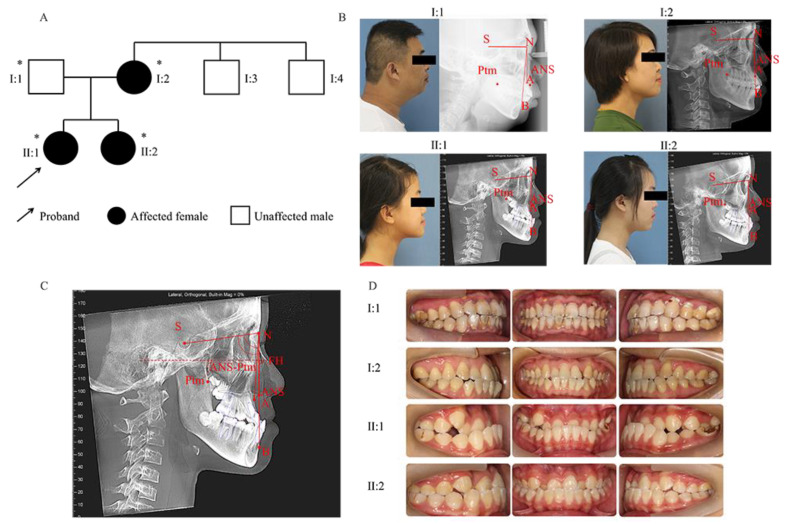
Pedigree and characterization of the skeletal Class III malocclusion. (**A**) Pedigree of the family. Asterisks represent participating family members. The proband (II:1) is a 12-year-old girl with skeletal Class III malocclusion. The arrow indicates the proband. (**B**) Profiles and lateral cephalograms. (**C**) Cephalometric landmark points. FH: Frankfort horizontal plane; S: sella; N: nasion; A: subspinale; B: supramental, Ptm: pterygomaxillary fissure; ANS: anterior nasal spine. (**D**) Intraoral views of the family members. I:2, II:1, and II:2 all have similar phenotypes.

**Figure 2:**
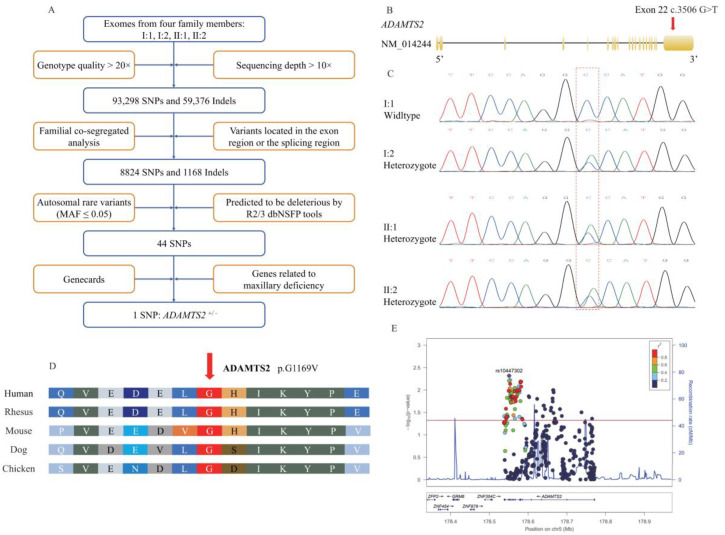
(**A**) Flow chart outlining selection of the causative variant. (**B**) Schematic diagram of the *ADAMTS2* gene showing the localization of the variant identified in this pedigree. (**C**) Sanger sequencing validation of the heterozygous c.3506G>T variant in the *ADAMTS2* gene shows individuals I:2, II:1, and II:2 are heterozygous and I:1 is wild type. Red dotted frame indicates the position of causative variants. (**D**) Conservation of each amino acid residue across species is shown. The red arrow indicates the mutated amino acid. Glycine at position 1169 is conserved. (**E**) Regional plot of the association between *ADAMTS2* and maxillary deficiency by logistic regression analysis. *x*-axis is the physical position in Mb with arrows denoting genes and expressed sequence tags in the region, the left *y*-axis plots the –log (*p*-value), and the right *y*-axis plots the CEPH recombination rate. The red horizontal line represents *p* = 0.05.

The proband, mother, and sister were diagnosed with skeletal Class III malocclusion and maxillary deficiency through clinical examination (Table 1, Figure 1). None of them had bad habits that can lead to skeletal Class III malocclusion. Therefore, to explore genetic factors related to maxillary deficiency, WES analysis in I:1, I:2, II:1, and II:2 identified a total of 152,674 unique variants after quality control and 9992 unique variants located in exon or splicing regions after familial segregation analysis. Subsequently, single nucleotide polymorphisms (SNPs) in the exonic and splice site region with minor allele frequency (MAF) > 0.05 were removed. SNPs remaining were analyzed by in silico prediction tools and predicted to be pathogenic by at least two dbNSFP tools (SIFT, Mutation Taster, PolyPhen-2). Forty-four low-frequency autosomal SNPs remained. Among these SNPs, a heterozygous missense variant (NM_014244: c.3506G>T) in exon 22 of *ADAMTS2* was identified to be associated with maxillary deficiency (Figure 2A, B).

Segregation testing by Sanger sequencing in the affected family members (I:2, II:1, and II:2) confirmed the c.3506G>T variant as heterozygous while the unaffected father was wildtype (Figure 2C). This variant leads to a glycine-to-valine substitution (NP_055059: p.G1169V) in an evolutionarily conserved DNA-binding domain (Figure S1). The SIFT score was 0.040 (deleterious), the Mutation Taster score was 0.959 (deleterious), and the PolyPhen-2 score was 0.679 (possibly damaging), which indicated that the variant was related to skeletal Class III malocclusion and maxillary deficiency. Moreover, this G residue and the amino acid are relatively conserved across 100 vertebrates by multiple-sequence alignment of *ADAMTS2* (Figure 2D). Additionally, the Z-score for the *ADAMTS2* was 1.43 in gnomAD (v2.1.1), which suggested that the transcript was intolerant of missense variation. The pLI (probability of being loss-of-function intolerant) scores for the *ADAMTS2* was 0.97, which indicated more intolerance to protein-truncating variation and haploinsufficiency (where heterozygous protein-truncating variants were not tolerated) [13,14].

Based on the cohort study, 500 variants in *ADAMTS2* (Chr5: 178,537,852-178,772,431) remained after quality control and 89 genetic variants indicated statistical significance after FDR correction (Table S2 and Figure 2E). These results demonstrated that *ADAMTS2* was associated with maxillary development.

### 2.2. Expression of the Mutation of ADAMTS2 in HEK293 and Proband-Derived Dental Pulp Stem Cells (DPSCs)

To evaluate the effect of the *ADAMTS2* variant on the protein level, Western blot of HEK293 cells with the wildtype and mutant allele was performed and a reduced level of ADAMTS2 was identified in the cells with the mutation (Figure S2A). Additionally, as neural crest cells (NCCs) participate in skull formation [15], and dental pulp stem cells (DPSCs) are derived from NCCs and thus differentiate into odontoblasts, osteoblasts, and adipocytes [16], we obtained DPSCs from orthodontic extraction of premolars from the proband and a healthy control, which were verified by flow cytometry (Figure S2B,C). The expression of *ADAMTS2* was significantly decreased in the proband’s DPSCs compared with the healthy control (*p* < 0.001) (Figure S2D), providing further verification that the variant (c.3506G>T) may associate with skeletal Class III malocclusion.

### 2.3. Expression and Phenotype of ADAMTS2 during Development

The expression of *ADAMTS2* was gradually elevated in jaw development during mouse embryonic days 10.5–14.5 (Figure S2E) and increased in the osteogenic process of human adult mesenchymal stromal cells (hMSCs) (Figure S2F). *ADAMTS2* is associated with abnormal facial development, such as midface retrusion and thin upper lip vermilion based on GeneCards (www.genecards.org (accessed on 17 December 2019)) [17] and DECIPHER (http://decipher.sanger.ac.uk/ (accessed on 1 May 2021)) [18]. In addition, *Adamts2*-knockout mice showed abnormal craniofacial development such as abnormal molar morphology, a triangular face, and short snout from Mouse Genome Informatics (MGI, http://www.informatics.jax.org/ (accessed on 1 May 2021)).

### 2.4. Adamts2 Deficiency Results in Zebrafish Craniofacial Defects

**Figure 3:**
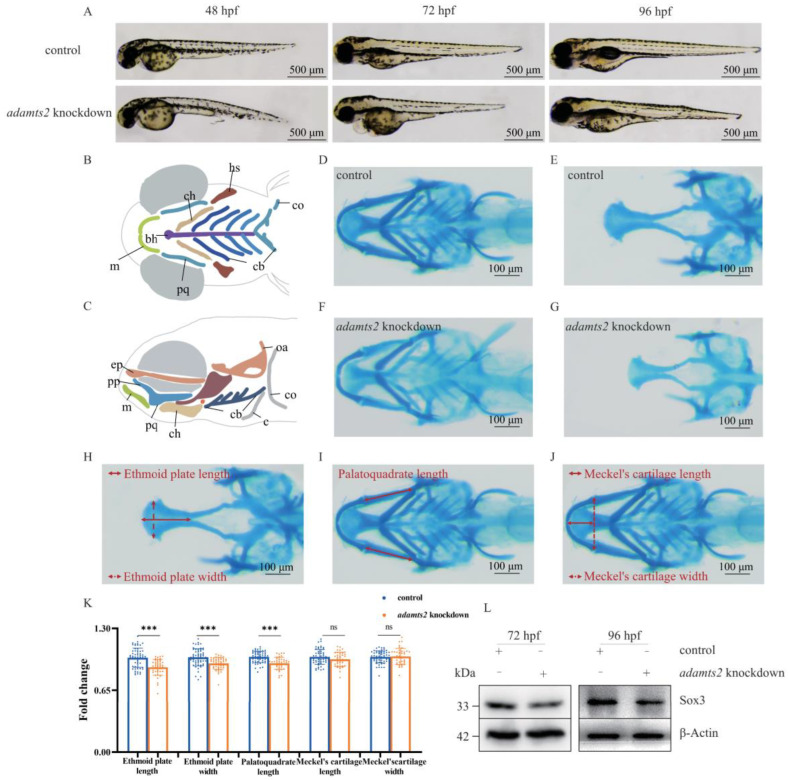
Phenotype in *adamts2*-knockdown zebrafish. (**A**) Zebrafish larvae were imaged in lateral positions with transmitted light at 48 hpf, 72 hpf, and 96 hpf. (**B**,**C**) Schematic diagram of zebrafish craniofacial structure from ventral view and lateral view: (bh) basihyal; (ch) ceratohyal; (co) coracoids of pectoral girdle; (cb) ceratobranchial; (c) cleithrum; (ep) ethmoid plate; (hs) hyosymplectic; (m) Meckel’s cartilage; (oa) occipital arch; (pp) pterygoid process of the palatoquadrate; (pq) palatoquadrate. (**D**,**E**) Control and (**F**,**G**) *adamts2*-knockdown zebrafish at 144 hpf were stained with Alcian blue to observe craniofacial cartilage structures from ventral and dorsal view. The red double arrow represents the measurement method of related indicators. (**H**) Ethmoid plate length and width; (**I**) palatoquadrate length; (**J**) Meckel’s cartilage length and width. (**K**) Scatter histogram showing change of palatoquadrate length, as well as length and width with respect to Meckel’s cartilage and ethmoid plate in control and *adamts2*-knockdown zebrafish. (**L**) Comparison of the sox3 protein expression levels in control crispant and *adamts2* crispant zebrafish at 72 hpf and 96 hpf. n = 3 independent replicates for all experiments. For (**D**–**K**): control crispant n = 56 embryos, *adamts2* crispant n = 41 embryos. Error bar represents the SD. ns, no significance, *** *p* < 0.001. Scale bars: 500 μm (**A**), 100 μm (**D**–**J**).

To explore the role of *ADAMTS2* in craniofacial development, a zebrafish model with CRISPR/Cas9-based targeted *adamts2*-knockdown was generated. The *adamts2*-knockdown embryos exhibited shorter body length, edema around the heart, and bent tail during early embryonic development (Figure 3A).

The ethmoid plate, palatoquadrate, and Meckel’s cartilage are the main components of zebrafish craniofacial cartilage, respectively analogous to human palate, the upper jaw, and the lower jaw [19] (Figure 3B,C). Alcian blue staining was performed to visualize cartilage (Figure 3D–G). The length and width of the ethmoid plate (Figure 3H) was shorter in the *adamts2*-knockdown group compared to the control group. Next, the length of palatoquadrate (Figure 3I) was also significantly decreased. However, lack of difference in the length and width of Meckel’s cartilage (Figure 3J) was observed between the two groups (Figure 3K).

Furthermore, Sox3, expressed in the ectoderm and could affect early embryonic craniofacial development [20], was downregulated in the *adamts2*-knockdown zebrafish model at 72 and 96 hours post fertilization (hpf) (Figure 3L).

### 2.5. ADAMTS2 Deficiency Disrupts EGFR Signaling

**Figure 4:**
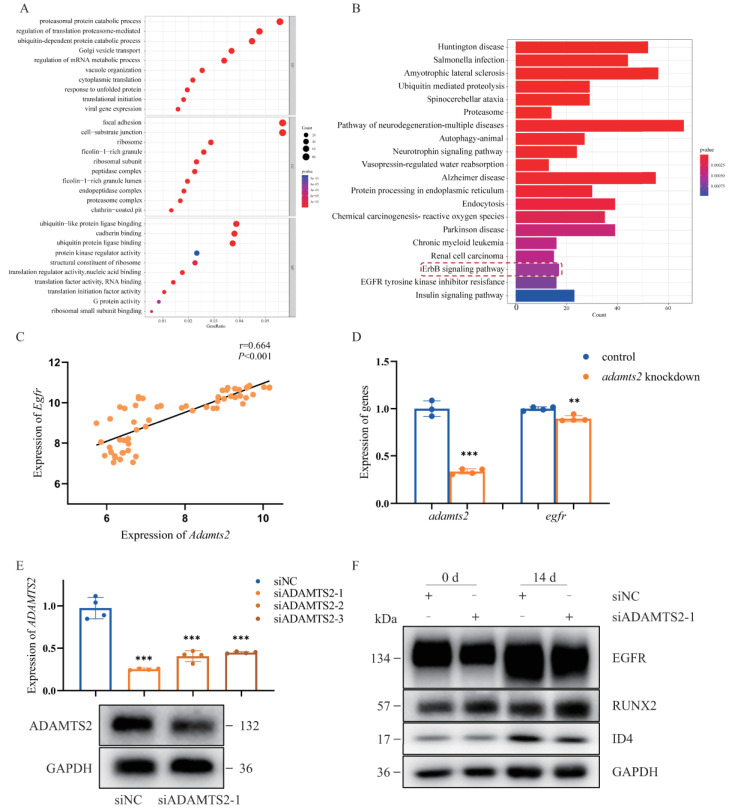
*ADAMTS2* was involved in several important pathological pathways based on gene ontology (GO) and Kyoto Encyclopedia of Genes and Genomes (KEGG) enrichment analysis. (**A**) The top 10 GO terms by gene ratio in the biological process terms and cellular component terms and molecular function terms in the enrichment analysis. (**B**) The top 20 most significantly enriched pathways by *p*-value with the genes co-expressed with *ADAMTS2* in the KEGG analysis. (**C**) *Egfr* was co-expressed with *Adamts2* during mouse embryonic development of upper and lower jaw (E10.5–E14.5). (**D**) qRT-PCR analysis of the expression of *egfr* in the *adamts2*-knockdown zebrafish embryos at 72 hpf. (**E**) qRT-PCR and Western blot analysis of the *ADAMTS2* knockdown efficiency in hMSCs. (**F**) Western blot analysis of the expression of pathway key genes and osteogenic indicators in hMSCs. Bars represent group means. ** *p* < 0.01, *** *p* < 0.001. n = 4 independent replicates for all experiments.

Gene ontology (GO) analysis showed that the genes co-expressed with *ADAMTS2* in the osteogenic process of hMSCs were mainly related to molecular function and biological processes including cell metabolism and cell adhesion (Figure 4A), while one of the most significant pathways identified in Kyoto Encyclopedia of Genes and Genomes (KEGG) analysis was the ErbB signaling pathway (Figure 4B). Moreover, *Egfr*, a key gene in the ErbB signaling pathway, was moderately and significantly correlated with *Adamts2* during mouse craniofacial development (Figure 4C). Thus, we detected the expression of *egfr* in zebrafish embryos and found it was also significantly down-regulated in the *adamts2*-knockdown zebrafish embryos compared with the control group (Figure 4D), which supported the previous bioinformatics results.

To explore the function of *ADAMTS2*, three siRNAs were transfected in hMSCs to knock down the expression of *ADAMTS2*. The siADAMTS2-1 has the highest efficiency in the RNA expression and could effectively knock down the level of protein (Figure 4E). Previous studies demonstrated that EGFR signaling stimulated osteoblast proliferation and inhibited differentiation [21], therefore, the level of proteins related to osteogenesis and the ErbB pathway were assayed by Western blot on day 14 after osteogenic induction. While the level of RUNX2, an osteogenic-related gene [22], has no change during the process of osteogenesis, the level of ID4, a member of Id protein family, was significantly downregulated. Also, the level of EGFR, a key gene in the ErbB signaling pathway, was also decreased (Figure 4F). Since ID4 was able to promote proliferation of early osteoblast progenitor cells and regulate the terminal differentiation of osteoblasts [23,24], suppression of EGFR signaling by ADAMTS2 may regulate cell differentiation during the osteogenic process of hMSCs.

## 3. Discussion

Skeletal Class III malocclusion is a severe abnormality in craniofacial development with genetic predisposition. Maxillary deficiency is an important type of skeletal Class III malocclusion, mainly manifesting as a depression in the middle of the face.

In this study, we identified a missense variant c.3506G>T in *ADAMTS2* and hypothesized that it was responsible for skeletal Class III malocclusion with maxillary deficiency in a Han Chinese family. The amino acid corresponding to the mutation from the pedigree was conserved among most species and predicted as deleterious. Furthermore, a replication cohort was recruited and dozens of genetic variants in *ADAMTS2* were associated with maxillary under-development.

*Adamts2* was consistently expressed in mouse jaws. Abnormal craniofacial development, such as abnormal molar morphology, triangular face, and short snout, has been shown in *Adamts2*-knockout mice. Additionally, DECIPHER showed that lack of *ADAMTS2* could result in abnormal facial development, such as midface retrusion and thin upper lip vermilion. Moreover, the expression of *ADAMTS2* was reduced with the mutation in HEK293, which was verified in DPSCs from the proband and a healthy control. This indicated that low expression of *ADAMTS2* may lead to craniofacial anomalies and maxillary deficiency.

Zebrafish is a well-suited animal model for studying craniofacial development [25]. Therefore, we generated a zebrafish model to explore the relationship between *adamts2* and craniofacial development. In the *adamts2*-knockdown zebrafish model, the body length and ethmoid plate length, as well as width and length of the palatoquadrate were significantly reduced compared to controls. The phenotype in the zebrafish shared similarity with that of human, indicating that *ADAMTS2* may affect the development of the upper jaw.

Subsequently, we explored the downstream mechanisms in which *ADAMTS2* may be involved in craniofacial development. Bioinformatics analysis and experiments in vitro showed that *ADAMTS2* played an important role in the process of osteogenesis via the ErbB signaling pathway which had vital impacts on osteoblast differentiation and anabolic bone metabolism [21,26,27]. As a member of ErbB signaling pathway and a necessary condition for chondrocyte maturation [28], *egfr* was suppressed in the *adamts2*-knockdown zebrafish, and positively correlated with *Adamts2* expression. *Egfr* can regulate the development of craniofacial bones and teeth and its abnormal expression may cause elongated snout, underdeveloped jaw, and a high incidence of cleft palate in mice [29]. Taken together, decreased level of ADAMTS2 may cause abnormal activation of the ErbB pathway to inhibit osteogenesis.

RUNX2 is also related to skeletal Class III malocclusion [30], however, after knocking down ADAMTS2 in hMSCs, ID4, which regulates the terminal differentiation of osteoblasts rather than RUNX2, was significantly downregulated during osteogenesis. These results suggested that there were two different mechanisms leading to skeletal Class III malocclusion between RUNX2 and ADAMTS2, which provided new clues for the etiology of skeletal Class III malocclusion.

In conclusion, our study identified a missense variant (NM_014244: c.3506G>T) in exon 22 of *ADAMTS2* associated with susceptibility to skeletal Class III malocclusion and maxillary deficiency and proved that *ADAMTS2* may be related to craniofacial development in vitro and in vivo. However, WES analysis was just based on a two-generation family with a mild phenotype of maxillary deficiency, which may lead to reduced accuracy of variant screening. In the cohort study, as a small population sample, false positives may be possible, which is also a limitation of this study. Further studies are required to replicate our findings.

## 4. Materials and Methods

### 4.1. Family and Cohort Recruitment

A Han Chinese family diagnosed with skeletal Class III malocclusion and maxillary deficiency was recruited. The diagnostic criteria of skeletal Class III malocclusion with maxillary deficiency were via clinical examination (excluding syndromes), medical history (excluding bad habits like chronic mouth breathing), cephalometric analysis (according to reference cephalometric values like ANB, SNA, SNB, and ANS-Ptm for Chinese) [12], lateral profile photographs, and intraoral examination. The female proband, mother, and sister also had a similar phenotype (Figure 1).

In the cohort, 114 patients with maxillary deficiency (SNA < 78.8°) and 696 controls with normal maxilla (78.8° ≤ SNA ≤ 86.8°) at the Affiliated Stomatology Hospital of Nanjing Medical University were recruited. Exclusion criteria of cohort included: (1) patients with syndromes; (2) congenital missing teeth; (3) supernumerary teeth; (4) or age of less than 12 years (Table S1).

Ethical approval for the study was obtained from the Ethics Review Committee on Human Research of the Affiliated Stomatology Hospital of Nanjing Medical University. Informed consent for participation in the study was obtained from each participant. Peripheral venous blood samples (2 mL) were collected, and genomic DNA was extracted using the Qiagen Blood Kit (Qiagen, Hilden, Germany) according to manufacturer’s instructions.

### 4.2. WES, Causative Variant Screening, Sanger Analysis, Species Conservation Analysis, Protein Structure Prediction, and Tolerance Prediction

Whole exome capture on the genomic DNA samples of I:1, I:2, II:1, and II:2 was performed using the Agilent SureSelect Human All Exon V6, followed by next-generation sequencing on the Illumina HiSeq sequencing platform. FASTQ files were aligned to the human GRCh37/hg19 reference genome using Burrows Wheeler Aligner software [31], and variant analyses were performed using Genome Analysis Toolkit (GATK, version 3.3.0) [32].

Multi-sample variant calling was performed by HaplotypeCaller (GATK version 3.3.0), and variants were filtered by Variant Quality Score Recalibration for both SNPs and insertions/deletions (InDels) including: (1) read depth (>10×) and genotype quality (>20×); (2) familial segregation analysis; (3) removal of ExAC, 1000 Genomes Project, Exome Sequencing Project (ESP6500) variants with MAF > 0.05 in autosomal chromosomes; (4) characterization of coding and non-coding SNVs within 14 bp of the splice site, and subsequent removal of SNPs predicted to be neutral by at least two dbNSFP tools (SIFT, MutationTaster, PolyPhen-2); and (5) identifying the potential causative gene related to maxillary deficiency which was predicted by GeneCards (www.genecards.org (accessed on 17 December 2019)) [17] (Figure 2A).

Sanger sequencing, with primers listed in Table S3, was applied to the candidate variant. The Sanger sequencing data were analyzed using Chromas (version 1.0.0.1, Technelysium Pty Ltd., South Brisbane, Australia). The amino acid corresponding to the SNP was assessed for evolutionary conservation using the UCSC genome browser (https://genome.ucsc.edu/ (accessed on 8 October 2021)). The modeling of ADAMTS2 was obtained from the AlphaFold Protein Structure Database (www.alphafold.ebi.ac.uk/ (accessed on 1 May 2022)) and the three-dimensional structural analysis was performed using PyMOL software (DeLano Scientific LLC, San Carlos, CA, USA). Z-score and pLI scores were calculated by gnomAD (v2.1.1) and reflected the tolerance of *ADAMTS2* to mutation [13,14].

### 4.3. Genotyping, Data Processing, Quality Control (QC) and Variant Selection

To further confirm the relationship between genetic variants in *ADAMTS2* and maxillary development, 114 cases with maxillary deficiency (SNA < 78.8°) and 696 controls with normal maxillary development (78.8° ≤ SNA ≤ 86.8°) were genotyped by Global Screening Array version 1.0 for logistic regression analysis (Table S1). Experiments were performed following the manufacturer’s protocol. The variants and samples with missing rate of >5% were filtered and SNPs that significantly deviated from Hardy–Weinberg equilibrium (HWE) (HWE < 1.0 × 10^−5^) were excluded. Multiple testing correction was performed using Benjamini–Hochberg FDR method, and the SNPs with FDR < 0.05 were retained.

### 4.4. Cell Culture, Plasmid and Small Interference RNA (siRNA) Constructs, and Transfection

Human embryonic kidney 293 (HEK293; ATCC^CRL−1573^) cells were purchased from ATCC and cultured in Eagle’s minimum essential medium (EMEM) with 10% fetal bovine serum, 1% penicillin–streptomycin solution at 37 °C in 5% CO_2_.

Premolars from orthodontic extraction obtained from the proband and a healthy control (female, age: 12) were split. Pulp tissues were minced, digested with collagenase type I (Item#: 1904MG100, BioFroxx, Germany), and trypsinized in alpha-modified Eagle’s medium (α-MEM) in a centrifuge tube with shaking every 5 min for four times total, and then the samples were collected in the medium-sized dish. The third generation of DPSCs were harvested and incubated with the antibodies published in Table S4 for 1 h in the dark and washed twice with PBS. The specific fluorescence of the samples was examined with a flow cytometer (BD Biosciences, San Jose, CA, USA).

The bone tissue removed during orthognathic surgery were collected to extract hMSCs and gently rinsed several times with α-MEM to eliminate residual oral fluids and blood. We used a surgical blade to release marrow contents into culture dishes containing α-MEM and centrifuged for 10 min at 1000 rpm/min.

DPSCs and hMSCs were cultured in α-MEM supplemented with 10% fetal bovine serum and 1% penicillin–streptomycin solution after centrifugation and resuspension. In the process of osteogenic induction, hMSCs were cultured with osteogenic cocktail (100 μM l-ascorbic acid, 10 mM β-glycerophosphate, and 10 nM dexamethasone) for up to 14 days, and the medium was replaced every other day.

The full-length coding region of the human *ADAMTS2* gene was cloned into the pcDNA3.1 vector to synthesize the wild-type plasmid. The mutated plasmid (G1169V) was generated by site-directed mutagenesis in vitro. All plasmids were synthesized by the Nanjing Genebay Institute (Genebay, Nanjing, China), and the entire sequence of the mutated constructs was confirmed by Genebay. The siRNA oligonucleotides were designed and purchased from GenePharma (Shanghai, China) (sequences are in Table S3). The plasmids with 100 ng final concentration and 100 nM siRNAs were transfected into HEK293 and hMSCs with Lipofectamine 2000 (Invitrogen, Carlsbad, CA, USA) following the manufacturer’s instructions.

### 4.5. Zebrafish Models

As *adamts2* in zebrafish is homologous to human (amino acid sequence similarity: 52.4%), the CRISPR/Cas9 method was used to knock down *adamts2* to generate a zebrafish model for craniofacial development. Mutagenesis was carried out using the *adamts2* primers shown in Table S3. The zebrafish model was generated according to a previously described method [33]. A total of 400 zebrafish embryos were randomly and equally assigned to experimental group and control group during microinjection. Dead and extremely deformed zebrafish embryos were excluded. Embryos were imaged using Nikon SMZ800N Stereomicroscope (NIKON Corporation, Tokyo, Japan).

All zebrafish (Tuebingen) were maintained according to established standards [34]. All experiments were performed in compliance with the Animal Ethics Committee of the Affiliated Stomatology Hospital of Nanjing Medical University.

### 4.6. Alcian Blue Staining

To detect morphological changes in craniofacial structures, 144 hpf zebrafish embryos were collected randomly and fixed in 95% ethanol overnight. After staining overnight with 0.02% Alcian blue (A8140, Solarbio, Beijing, China), the samples were soaked in distilled water for 10 min and bleached with 1.5% H_2_O_2_/1.5% KOH for 3 h until soft tissue became transparent. Finally, the specimens were stored in 50% glycerol at room temperature. Phenotypes were quantitatively analyzed by evaluating the length/width of ethmoid plate, palatoquadrate, and Meckel’s cartilage [35,36].

### 4.7. RNA Extraction and Quantitative Real-Time PCR (qRT-PCR)

RNA extraction and qRT-PCR were performed on DPSCs, hMSCs, and zebrafish embryos at 72 hpf according to a previously described method [37]. PCR amplification was performed using a primer pair shown in Table S3. All data were analyzed using the 2^−ΔΔCt^ method.

### 4.8. Western Blot

Western blot was performed on the following materials according to a previously described method [38]: HEK293 cells which included an empty vector transfection control, wildtype and mutation-containing plasmids, hMSCs which were transfected by siRNA at day (d) 0 and 14, and zebrafish embryos at 72 hpf and 96 hpf. Primary antibodies were included in Table S4.

### 4.9. Co-Expression, Correlation, and Pathway Enrichment Analysis

Based on the expression values for osteogenesis of hMSCs at 0, 1, 3, and 7 d (GSE18043) [39], the correlation was calculated between *ADAMTS2* and other genes, which were detected by Affymetrix HG-U133 Plus 2.0 arrays. All genes detected in the gene expression array have been used as a background list to adjust detection bias [40]. Significant genes (Pearson’s coefficient > 0.8 and *p* < 0.05) were identified for GO analysis and KEGG pathways analyses. Enrichment analysis was conducted using the R package “cluster-Profiler”. Additionally, the correlation between *ADAMTS2* and genes in the pathway was evaluated based on the expression value in mouse embryonic craniofacial tissues from the FaceBase consortium (http://www.facebase.org/ (accessed on 1 June 2021) GSE67985) [41].

### 4.10. Statistical Analysis

The sample size of zebrafish embryos had been calculated considering the theoretical difference between the means and the theoretical size of the standard deviation. The embryos were randomly assigned to injection without operator blinding. Logistic regression analysis was performed between those with maxillary deficiency and controls with normal maxillary development by PLINK software (version 1.07). Co-expression analysis, GO, and KEGG analyses were performed using R software (version 4.0.5), and false discovery rate (FDR) was used to control for multiple testing. Statistical analyses in all graphs were performed using a two-tailed, unpaired Student’s *t*-test (GraphPad Prism-6 software; San Diego, CA, USA). Data were considered statistically significant at *p*-values < 0.05.

## Figures and Tables

**Table 1:** Summary of measurements in all individuals.

Cephalometric Variables	I:1	I:2	II:1	II:2	Norms *
ANB(°)	1.2	−1.3	−3.6	−3.1	2.7 ± 2.0
SNA(°)	81.8	78.3	78.3	78.4	82.8 ± 4.0
SNB(°)	80.6	79.5	81.9	81.5	80.1 ± 3.9
ANS-Ptm (mm)	52.0	42.9	42.3	43.1	Male permanent dentition: 52.1 ± 2.8 Female permanent dentition: 49.9 ± 2.1 Early female permanent dentition: 47.7 ± 2.9

* Cephalometric standards of Chinese [12]; ANB, anteroposterior relationship of the maxilla and mandible; SNA, anteroposterior maxillary position to anterior cranial plane; SNB, anteroposterior mandibular position to anterior cranial plane; ANS-Ptm, length of maxillary.

## Data Availability

Microarray results are derived from secondary analysis of two previously published datasets, which can be accessed via NCBI’s Gene Expression Omnibus using accession numbers GSE18043and GSE67958. Raw data from this study are available upon reasonable request to the corresponding author.

## Supplementary Materials

The following supporting information can be downloaded at: https://www.mdpi.com/article/10.3390/ijms231810673/s1.
