# Supplementary material for: Skeletal Class III Malocclusion Is Associated with ADAMTS2 Variants and Reduced Expression in a Familial Case
Source: Int J Mol Sci. 2022 Sep 14;23(18):10673. doi: 10.3390/ijms231810673 (PMC9505033; doi:10.3390/ijms231810673)
Supplement: Supplementary file 1 [file ijms-23-10673-s001.zip › ijms-1890671-supplementary.pdf]

Table S1. Characteristics of individuals in the cohort

Variables	Cases<br>SNA<78.8°	Controls<br>78.8°≤SNA≤86.8°	p^a
	N=114 (%)	*N*=696 (%)	
**Gender**			0.176
Male	29 (25.4%)	221 (31.8%)	
Female	85 (74.6%)	475 (68.2%)	
**Age**			0.726
(Mean±SD)	18.60±7.15	18.83±6.54	

Cephalometric standards of Chinese;

SNA, anteroposterior maxillary position to anterior cranial plane;

[a]*p* value of Chi-square test for heterogeneity.

Table S2. SNPs in *ADAMTS2* associated with SNA angle between maxillary deficiency and normal maxillary

Chr	SNP	Position	Alleles (minor/major)	MAF[&] (case/control)	OR (95%CI)	*p* value[*]	FDR[#]
5	rs10447302	178550869	(C/T)	0.38/0.29	1.54 (1.14-2.07)	0.005	0.025
5	rs6601018	178553341	(T/C)	0.40/0.51	0.67 (0.50-0.89)	0.006	0.025
5	rs888759	178565121	(C/T)	0.50/0.41	1.46 (1.10-1.93)	0.009	0.025
5	rs7718671	178552715	(G/A)	0.46/0.37	1.46 (1.10-1.94)	0.009	0.025
5	rs11741099	178664463	(G/A)	0.07/0.04	2.09 (1.19-3.65)	0.010	0.025
5	rs62396137	178601466	(A/G)	0.23/0.31	0.66 (0.48-0.92)	0.014	0.025
5	rs4701079	178623326	(A/G)	0.36/0.46	0.69 (0.51-0.93)	0.016	0.025
5	rs7718805	178639972	(A/C)	0.04/0.08	0.44 (0.22-0.89)	0.022	0.030
5	rs378074	178682067	(G/A)	0.09/0.06	1.77 (1.07-2.94)	0.027	0.042
5	rs79046216	178737618	(T/C)	0.06/0.03	2.03 (1.06-3.86)	0.032	0.041
5	rs3797615	178538472	(A/G)	0.39/0.47	0.74 (0.55-0.98)	0.039	0.043
5	rs138850872	178547013	(A/G)	0.04/0.02	2.43 (1.05-5.65)	0.039	0.043
5	rs26812	178764906	(A/G)	0.26/0.19	1.40 (1.01-1.95)	0.044	0.043
5	rs6871456	178550811	(C/G)	0.41/0.51	0.67 (0.51-0.89)	0.006	0.025
5	rs10076252	178580091	(A/G)	0.35/0.26	1.52 (1.13-2.06)	0.006	0.025
5	rs2278221	178581859	(A/G)	0.37/0.28	1.51 (1.12-2.04)	0.007	0.025
5	rs3776817	178579748	(T/C)	0.37/0.28	1.50 (1.12-2.02)	0.007	0.025
5	rs6601019	178553450	(G/A)	0.38/0.29	1.49 (1.11-2.00)	0.007	0.025
5	rs3797595	178575917	(T/C)	0.58/0.49	1.46 (1.10-1.92)	0.008	0.025
5	rs2278222	178581797	(T/C)	0.38/0.29	1.49 (1.11-2.01)	0.008	0.025
5	rs35944670	178564499	(T/C)	0.50/0.41	1.45 (1.10-1.93)	0.009	0.025
5	rs10036369	178570902	(T/C)	0.58/0.48	1.45 (1.10-1.91)	0.009	0.025
5	rs9687070	178569149	(A/G)	0.58/0.48	1.44 (1.09-1.91)	0.010	0.025
5	rs35814218	178577570	(T/C)	0.37/0.28	1.48 (1.10-1.98)	0.010	0.025

5	rs11746802	178665185	(T/C)	0.07/0.04	2.09 (1.19-3.65)	0.010	0.025
5	rs34132209	178572884	(T/G)	0.37/0.28	1.48 (1.10-1.98)	0.010	0.025
5	rs10065978	178575384	(A/G)	0.58/0.48	1.44 (1.09-1.90)	0.010	0.025
5	rs3822599	178576471	(C/T)	0.58/0.48	1.44 (1.09-1.90)	0.010	0.025
5	rs3756566	178576631	(A/G)	0.58/0.48	1.44 (1.09-1.90)	0.010	0.025
5	rs2411905	178577487	(A/G)	0.58/0.48	1.44 (1.09-1.90)	0.010	0.025
5	rs6860046	178552518	(A/G)	0.45/0.36	1.46 (1.09-1.95)	0.010	0.025
5	rs2303641	178549791	(A/G)	0.37/0.29	1.49 (1.10-2.01)	0.010	0.025
5	rs71596406	178561891	(C/A)	0.44/0.35	1.46 (1.09-1.95)	0.011	0.025
5	rs34447103	178565237	(T/C)	0.37/0.29	1.47 (1.09-1.97)	0.011	0.025
5	rs7727517	178573954	(T/C)	0.42/0.51	0.70 (0.53-0.92)	0.011	0.025
5	rs35926292	178565543	(G/C)	0.37/0.29	1.47 (1.09-1.98)	0.011	0.025
5	rs34346845	178571507	(T/C)	0.37/0.29	1.47 (1.09-1.98)	0.011	0.025
5	rs469262	178665824	(G/A)	0.08/0.04	2.03 (1.17-3.50)	0.011	0.025
5	rs2411907	178577267	(G/C)	0.41/0.51	0.69 (0.52-0.92)	0.011	0.025
5	rs3776816	178574868	(G/A)	0.58/0.49	1.43 (1.08-1.89)	0.011	0.025
5	rs3776815	178574732	(A/C)	0.58/0.49	1.43 (1.08-1.89)	0.011	0.025
5	rs7722275	178557919	(T/C)	0.58/0.49	1.43 (1.08-1.89)	0.012	0.025
5	rs4701054	178553931	(G/A)	0.41/0.51	0.70 (0.53-0.93)	0.012	0.025
5	rs4701057	178571857	(A/G)	0.50/0.41	1.43 (1.08-1.90)	0.013	0.025
5	rs35648285	178555768	(A/G)	0.37/0.29	1.45 (1.08-1.95)	0.014	0.025
5	rs12659833	178553924	(C/T)	0.46/0.37	1.43 (1.08-1.90)	0.014	0.025
5	rs62396128	178561707	(G/A)	0.37/0.29	1.45 (1.08-1.95)	0.014	0.025
5	rs58887996	178569941	(A/T)	0.37/0.29	1.45 (1.08-1.95)	0.014	0.025
5	rs2303645	178557292	(T/C)	0.37/0.29	1.45 (1.08-1.95)	0.014	0.025
5	rs7722433	178558025	(T/C)	0.37/0.29	1.45 (1.08-1.95)	0.014	0.025
5	rs34211544	178558183	(G/A)	0.37/0.29	1.45 (1.08-1.95)	0.014	0.025
5	rs7704524	178558194	(G/A)	0.37/0.29	1.45 (1.08-1.95)	0.014	0.025

5	rs10464082	178561064	(A/G)	0.37/0.29	1.45 (1.08-1.95)	0.014	0.025
5	rs873987	178602683	(A/G)	0.23/0.31	0.67 (0.48-0.92)	0.015	0.025
5	rs3776814	178562644	(G/A)	0.45/0.36	1.42 (1.07-1.89)	0.015	0.025
5	rs4701049	178545214	(C/G)	0.36/0.28	1.47 (1.08-2.00)	0.015	0.025
5	rs6861255	178563895	(C/T)	0.45/0.36	1.42 (1.07-1.88)	0.015	0.025
5	rs34389865	178558053	(C/G)	0.37/0.29	1.44 (1.07-1.94)	0.015	0.025
5	rs7722465	178558088	(T/C)	0.37/0.29	1.44 (1.07-1.94)	0.015	0.025
5	rs467017	178663005	(T/G)	0.08/0.04	1.96 (1.14-3.38)	0.016	0.025
5	rs469290	178663225	(C/T)	0.08/0.04	1.96 (1.14-3.38)	0.016	0.025
5	rs2303638	178567054	(G/A)	0.37/0.29	1.44 (1.07-1.93)	0.016	0.025
5	rs2303643	178553185	(G/A)	0.49/0.40	1.42 (1.07-1.90)	0.017	0.025
5	rs3776818	178579914	(C/T)	0.50/0.41	1.41 (1.06-1.86)	0.018	0.025
5	rs4701049	178545214	(G/T)	0.36/0.28	1.38 (1.02-1.86)	0.035	0.025
5	rs35311590	178567925	(T/C)	0.37/0.29	1.43 (1.06-1.92)	0.018	0.027
5	rs469568	178663408	(C/A)	0.08/0.04	1.93 (1.12-3.32)	0.018	0.027
5	rs3776810	178556385	(C/T)	0.45/0.36	1.41 (1.06-1.87)	0.019	0.027
5	rs2251105	178557142	(G/A)	0.45/0.36	1.41 (1.06-1.87)	0.019	0.027
5	rs3776811	178561267	(C/G)	0.45/0.36	1.41 (1.06-1.87)	0.019	0.027
5	rs9286043	178563362	(C/T)	0.45/0.36	1.41 (1.06-1.87)	0.019	0.027
5	rs7722105	178557841	(G/C)	0.45/0.36	1.41 (1.06-1.87)	0.019	0.027
5	rs6601020	178555463	(A/G)	0.45/0.36	1.40 (1.06-1.86)	0.019	0.027
5	rs466750	178640760	(C/A)	0.04/0.08	0.44 (0.22-0.89)	0.022	0.030
5	rs3822597	178565708	(A/C)	0.42/0.50	0.73 (0.55-0.96)	0.027	0.030
5	rs11739530	178659639	(A/G)	0.07/0.03	1.98 (1.09-3.59)	0.025	0.032
5	rs4700787	178620251	(T/C)	0.38/0.46	0.72 (0.54-0.97)	0.030	0.033
5	rs11744583	178643569	(G/A)	0.04/0.08	0.45 (0.23-0.91)	0.027	0.035
5	rs1109178	178658440	(T/G)	0.07/0.04	1.94 (1.07-3.52)	0.029	0.037
5	rs3776807	178542682	(G/A)	0.37/0.30	1.41 (1.04-1.90)	0.026	0.037

5	rs13165525	178665562	(A/G)	0.07/0.04	1.91 (1.06-3.46)	0.033	0.041
5	rs2411906	178577392	(C/T)	0.42/0.50	0.74 (0.56-0.99)	0.040	0.041
5	rs113250460	178561982	(A/G)	0.45/0.37	1.36 (1.02-1.80)	0.037	0.043
5	rs55849753	178544213	(A/G)	0.36/0.29	1.38 (1.02-1.88)	0.039	0.043
5	rs3797592	178565650	(T/C)	0.39/0.47	0.74 (0.55-0.99)	0.043	0.043
5	rs189256	178637965	(C/T)	0.04/0.08	0.49 (0.25-0.96)	0.039	0.045
5	rs4701050	178545385	(C/T)	0.43/0.36	1.36 (1.02-1.83)	0.039	0.045
5	rs7729756	178584302	(T/C)	0.35/0.28	1.38 (1.01-1.88)	0.044	0.045
5	rs34865831	178585187	(T/C)	0.35/0.28	1.38 (1.01-1.89)	0.046	0.045
5	rs173104	178686928	(C/T)	0.09/0.06	1.74 (1.03-2.92)	0.038	0.050
5	rs3797590	178544084	(A/G)	0.43/0.36	1.36 (1.01-1.82)	0.043	0.050
5	rs3776808	178542705	(A/G)	0.34/0.27	1.39 (1.01-1.91)	0.042	0.052
5	rs3776809	178542706	(G/A)	0.34/0.27	1.39 (1.01-1.91)	0.042	0.052
5	rs338875	178669297	(T/C)	0.09/0.06	1.69 (1.02-2.81)	0.044	0.057
5	rs12153128	178668714	(A/G)	0.09/0.06	1.69 (1.01-2.84)	0.047	0.058
5	rs4700789	178669361	(G/A)	0.09/0.06	1.69 (1.01-2.84)	0.047	0.058
5	rs338874	178668964	(C/G)	0.09/0.06	1.67 (1.00-2.77)	0.049	0.060
5	rs338876	178669870	(G/A)	0.09/0.06	1.67 (1.00-2.77)	0.049	0.060

[&]MAF， Minor Allele Frequency

[*] Logistic regression analysis

[#] Benjamini-Hochberg FDR

Table S3. Primer sequences

Application	Gene	Description	Forward
Sanger	*ADAMTS2*	Forward	5’-CCGAGCATCTCTTTCTTCCGCATCT-3’
		Reverse	5’-CGCCTGGGAAGCACAACGAC-3’
real-time PCR	*ADAMTS2*	Forward	5’-GACACGGGCCACGATGAATA-3’
		Reverse	5’-GGTGACAGGAGCATAGCCTT-3’
	GAPDH	Forward	5’-GGACCTGACCTGCCGTCTAG-3’
		Reverse	5’-GTAGCCCAGGATGCCCTTGA-3’
	adamts2	Forward	5'-GACAGGAGAGGAGGAAGGA-3'
		Reverse	5'-AAGACCACCCAGAGGAGAA-3'
	Egfr	Forward	5'-GTGCGTGCAGATCGCTAAAG-3'
		Reverse	5'-TTGACATGCTGAGGCGTCTT-3'
	Gapdh	Forward	5'-ATTGCCGTTCATCCATCTT-3'
		Reverse	5'-TGCCATCAGGTCACATACAC-3'
siRNA	siADAMTS2-1	Forward	5′- CAGGCAAGUUCAUCUUAAATT-3′
		Reverse	5′-UUUAAGAUGAACUUGCCUGTT-3′
	siADAMTS2-2	Forward	5′-GGCGACAAGUCAAUAUUCUTT-3′
		Reverse	5′- AGAAUAUUGACUUGUCGCCTT-3′
	siADAMTS2-3	Forward	5′-GUCUCACUGACGUACAAAUTT-3′
		Reverse	5′-AUUUGUACGUCAGUGAGACTT-3′
	siNC	Forward	5′-UUCUCCGAACGUGUCACGUTT-3′
		Reverse	5′-ACGUGACACGUUCGGAGAATT-3′
sgRNA	*adamts2*	Forward	5’-GCTGGTGAGGCCGGTCAGTG-3’
		Forward	5’-GGAGGAGGCAGGCCAACCAGCA-3’
		Forward	5’-GGACGATAACCAAACACA CT-3’
		Forward	5’-GGGTTATGTTACCAATGTGC-3’
		Reverse-common	5’-AAAAAAAGCACCGACTCGGTGCCAC-3’

Table S4. Summary of antibodies

Antibody	Dilution ratio	Corporation	Item No.
ADAMTS2	1:1000	Santa Cruz, USA	sc-393562,
EGFR	1:1000	abcam, Cambridge, UK	ab52894
ID4	1:1000	abcam, Cambridge, UK	ab220166
RUNX2	1:1000	Cell Signaling Technology, Boston, USA	#12556
GAPDH	1:1000	Beyotime, Shanghai, China	AG019
Sox3	1:1000	GeneTex, Irvine, CA, USA	#GTX132494
β-Actin	1:1000	GeneTex, Irvine, CA, USA	#GTX629630
CD29-APC	20μl	BD Pharmingen, England	561794
CD90-TITC	5μl	BD Pharmingen, England	555595
CD34-PE	5μl	BD Pharmingen, England	560941
CD45-PE	5μl	BD Pharmingen, England	560915

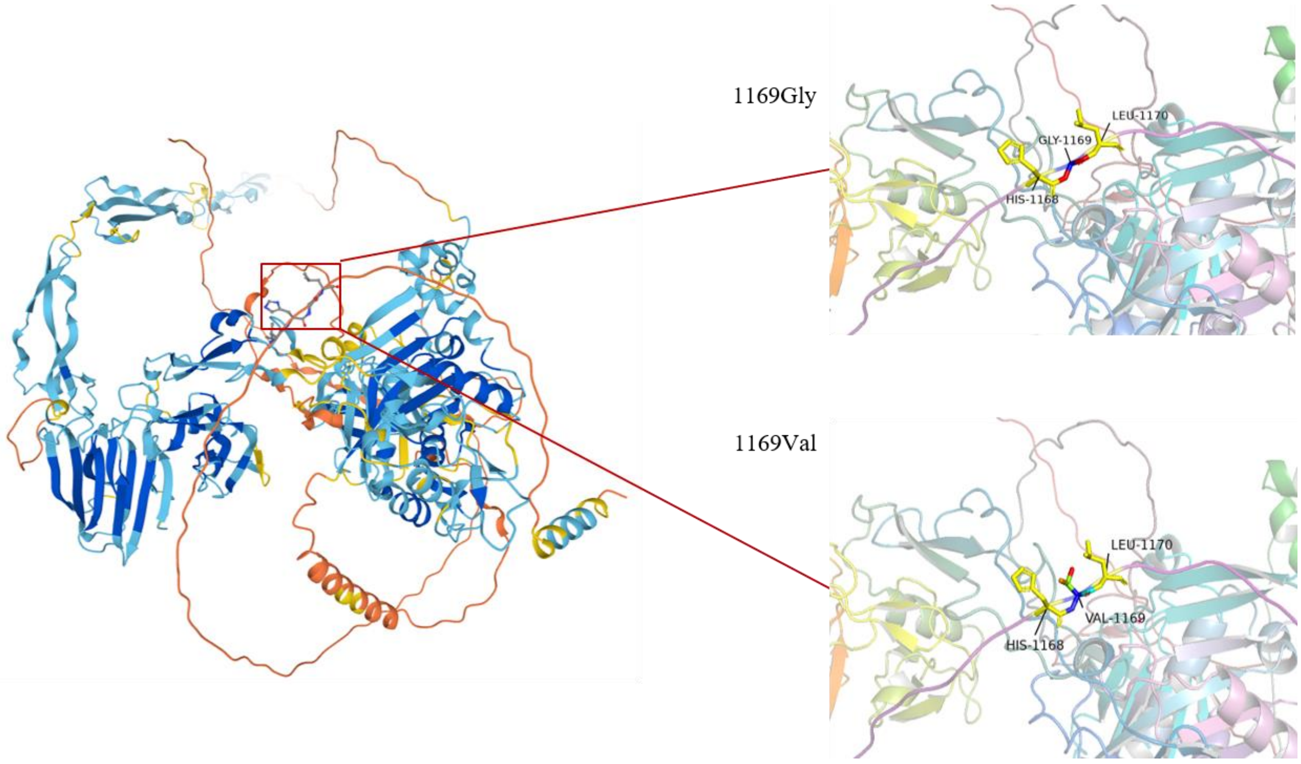

Fig S1. The predicted three-dimensional models of ADAMTS2 with wild-type 1169 residue (Gly) and mutated 1169 residue (Val).

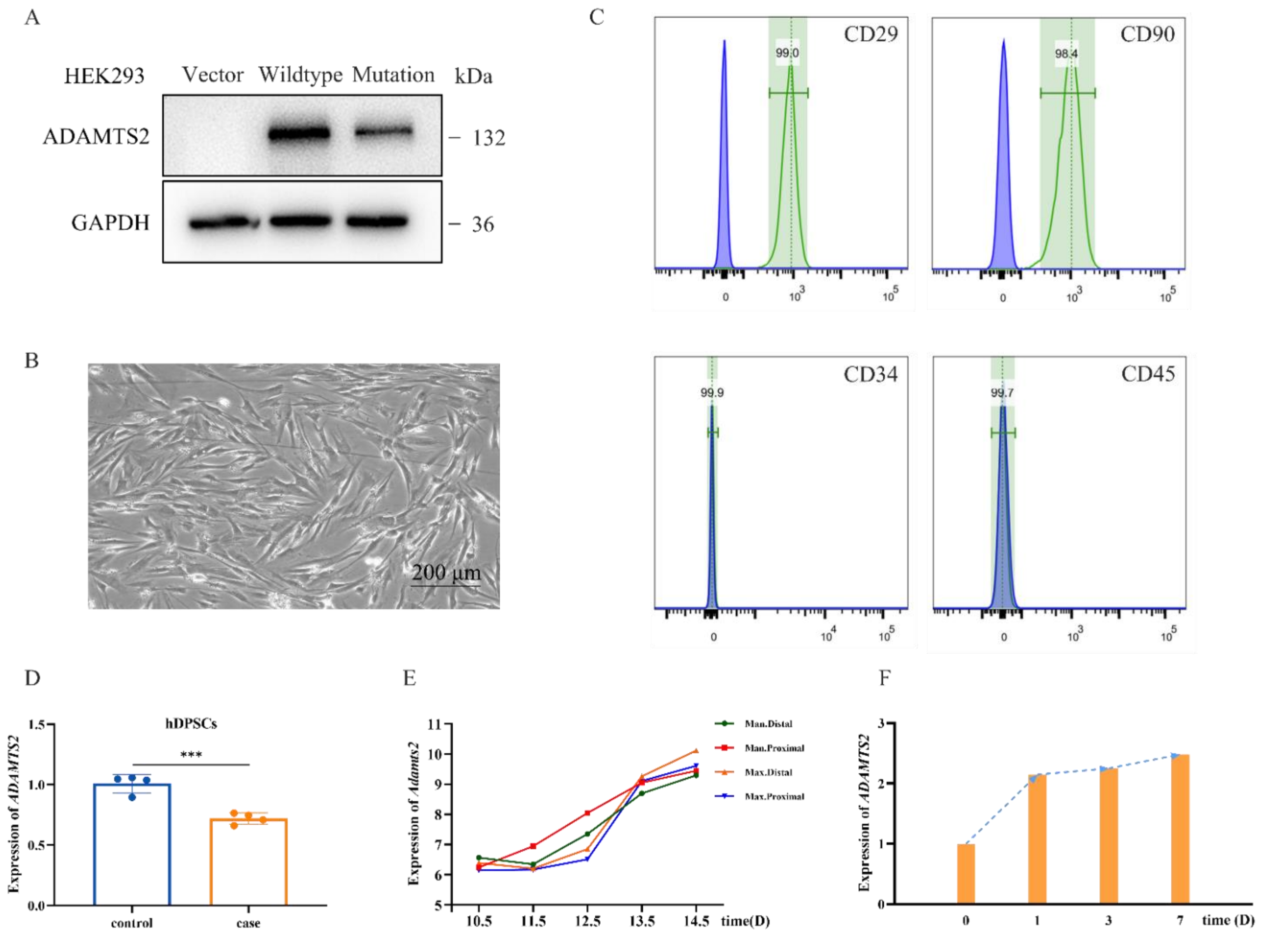

Fig S2. The expression of *ADAMTS2*. A. Western blot analysis of total protein from wildtype or mutated ADAMTS2 cells transfected into HEK293 cells with empty vector as a negative control. n=3 independent replicates for all experiments. B. The hDPSCs from II:1 is cultured to the third generation. Scale bars: 200 μm. C. Flow cytometric identification of hDPSCs surface markers shows stem cell characteristics. D. The expression of *ADAMTS2* is reduced in proband's DPSCs (***p<0.001); E. The expression of *Adamts2* changes dynamically in upper and lower jaw during embryonic (E10.5-E14.5) mouse development; F. The expression of *ADAMTS2* gradually increases during the osteogenesis of hMSCs.
